# Oceanic currents maintain the genetic structure of non-marine coastal taxa in the western Mediterranean Sea

**DOI:** 10.1038/s44185-023-00028-0

**Published:** 2023-11-20

**Authors:** Adrián Villastrigo, Víctor Orenes-Salazar, Antonio José García-Meseguer, Juana María Mirón-Gatón, Baptiste Mourre, Andrés Millán, Josefa Velasco

**Affiliations:** 1 Division of Entomology, SNSB-Zoologische Staatssammlung München, Münchhausenstraße 21, 81247 München, Germany; 2 Departamento de Ecología e Hidrología, Universidad de Murcia, 30100 Murcia, Spain; 3 Balearic Islands Coastal Observing and Forecasting System, 07121 Palma, Spain

**Keywords:** Biodiversity, Evolutionary ecology, Biogeography

## Abstract

Coastal habitats are amongst the most dynamic on Earth, due to their simultaneous exposure to terrestrial, oceanic and atmospheric processes. Coastal taxa are therefore often ecologically specialised and adapted to withstand frequent shifts in sea level, wave exposure, temperature or salinity. This specialisation often resulting in significant cryptic diversity. Previous molecular studies have suggested that genetic differentiation in non-marine coastal organisms may be influenced by oceanic currents and fronts, but the extent to which such processes affect dispersal and evolution of such taxa remains unclear. Here we explore whether population genetic structure in two supralittoral rockpool beetle species (genus *Ochthebius*) can be predicted from the general circulation pattern of the marine currents and associated oceanic fronts. We simulated dispersal using a Lagrangian particle tracking model and compared this with population genetic structure inferred from *COI* (mitochondrial) and *wingless* (nuclear) genes applying linear models and Mantel tests. We show that a biophysical model based on oceanic currents and fronts in the western Mediterranean Sea is a much better predictor of observed population genetic structure than isolation by distance in both species. Our results show that oceanic processes, besides shaping contemporary population connectivity in fully marine organisms, also exert a meaningful influence on terrestrially-derived coastal taxa such as supralittoral rockpool beetles — the first time this mode of dispersal has been demonstrated in an insect.

## Introduction

Coastlines are ecotones between land and ocean and are amongst the most dynamic environments on Earth. Within these areas, supralittoral rockpools are geographically restricted, fragmented and exposed to processes of physical disturbance operating across a broad variety of temporal and spatial scales. They suffer constant habitat modifications driven by several terrestrial, oceanic and atmospheric processes, and are highly vulnerable to large-scale physical disturbances^1^. The biological assemblages found in these areas are dominated by strongly adapted species that can withstand frequent shifts in sea level, temperature and salinity^2,3^. As a consequence, coastal taxa are often ecologically specialised, frequently restricted to specific habitats. These taxa may harbour significant cryptic diversity^4–7^ due to ecomorphological constraints and the isolation of populations by major physical coastal disturbances such as uplifts, tsunamis, hurricanes, glaciations and sea-level fluctuations^1^. Whilst between the tidelines, coastal biota is dominated by marine-derived species, the composition of supralittoral communities is very different. Such habitats are typically species-poor^8^, but support a suite of specialised, ecologically restricted taxa, many of which are terrestrial or freshwater in origin rather than marine^6^. Compared to intertidal biotas, the inhabitants of the supralittoral are much less well studied, and the extent to which their population biology and evolution resemble terrestrial or marine organisms remains unclear.

Although major disturbances play an important role in isolating populations, other mechanisms can also drive genetic differentiation. Habitat discontinuities, local adaptation, behaviour and dispersal capabilities are key to explaining the genetic variability between and within populations^9,10^. In the sea, it is widely recognised that oceanic fronts and currents represent sharp discontinuities of physical and biochemical variables^11^ and constitute barriers to faunal dispersal, often limiting gene flow between marine populations^12^. These oceanic features can explain the genetic structure of benthic marine littoral taxa, particularly those which are sedentary or with low vagility, in which population units are distributed in habitat patches whose connectivity is mediated by species’ life history traits^13,14^.

Despite comprehensive evidence for the influence of oceanic fronts and currents on the genetic structure of marine animals, their importance for organisms that are not strictly marine remains unclear, including the insects which dominate supralittoral rockpools in the terrestrial-marine ecotone. Beetles of the genus *Ochthebius* Leach, 1815 are the predominant macroinvertebrates inhabiting coastal supralittoral rockpools in many parts of the world, being particularly diverse in the western Palaearctic^15,16^. Although most adult *Ochthebius* can fly, their active dispersal capabilities may be limited by their small body size (c. 1–2 mm long) and erratic flight^17^. For this reason, similar to many small insects, they are likely to be subjected to passive dispersal by air currents or birds over short distances and by winds or even marine currents over medium to large spatial scale^18,19^. As previously proposed^7^, taxa such as *Ochthebius* may be affected by the action of storms and cyclones, which can promote gene flow among populations. These taxa lack a marine dispersal stage in their life cycle, but large waves or spring tides can remove them from supralittoral rockpools. Under these conditions, more resistant life-history stages such as eggs, able to survive at marine salinity conditions^20,21^, could be dispersed through coastal currents by floating or drifting on algal mats and plants/macroalgal remains, where they frequently oviposit. Marine currents may therefore be critical in shaping the diversity, connectivity and demography of inhabitants of supralittoral rockpools such as *Ochthebius* beetles, as reported in other arthropods such as Collembola^22^ and mites^23^.

On the western Mediterranean coast, the phylogeography of supralittoral *Ochthebius* has recently been studied^7^ using a combination of mitochondrial and nuclear genes. Villastrigo et al.^7^ proposed that both historical and contemporary marine barriers (Messinian Salinity Crisis and the Ibiza Channel, respectively) may have driven genetic isolation between extant populations. In particular, strong genetic discontinuities were observed in populations of two species (*Ochthebius quadricollis* Mulsant, 1844 and *Ochthebius subinteger* Mulsant & Rey, 1861) located on either side of the Ibiza Channel, which also acts as an impermeable geographical barrier for another (*Ochthebius lejolisii* Mulsant & Rey, 1861). Another genetic discontinuity was also found between western and eastern coastal areas of the Alboran Sea, where the interface between two anticyclonic gyres may operate as a barrier^24^. In a similar manner, *Ochthebius* populations from coastal rockpools in South Africa have apparently diverged in association with the Benguela and Agulhas marine currents^19^, like some fully marine members of the southern African rocky shore and estuarine communities^25^. Evidence suggests that properties of the ocean may exert a significant influence on the population biology and evolution of such taxa.

To investigate this further, and understand whether supralittoral rockpool inhabitants are primarily dispersed by oceanic currents, integrated approaches must include not only genetic data, but also biological and physical dispersal modelling (hereafter biophysical models). Such approaches have become common tools for the study of connectivity in both marine and terrestrial organisms^26^ due to the advantages they offer for estimating population connectivity over a range of spatial and temporal scales, and for multiple species at relatively low cost^14^. To date, they have been widely used in combination with genetic data to explore such questions in fully marine taxa^27,28^, but not the terrestrial-derived inhabitants of the coastal zone. In the Mediterranean Sea, they have also been used to assess levels of connectivity between protected and unprotected populations, evaluate dispersal potential from effective marine reserves, and hindcast the position of fish spawning areas^29–31^. Whilst biophysical models have proven to be relevant tools in the study of marine connectivity, they do need to be supported by other, complementary, techniques^32^. Combining oceanographic models with population genetic information^32^ is key to understanding how oceanographic currents and fronts may influence the extent of genetic variation within and among coastal populations. For example, strong links between genetic structure and biophysical model prediction would indicate that a significant part of the species’ phylogeographical structure results from ocean dynamics^33^.

This study is the first to combine population genetics and biophysical modelling to explore the possible role of oceanic currents and fronts in affecting the contemporary genetic structure of non-marine coastal organisms, focussing on supralittoral rockpool beetles in the Western Mediterranean Basin. Using this combined approach, we investigate whether the genetic structure of the studied species is best explained by a biophysical model or isolation by distance. We simulate potential population connectivity via marine propagules using a Lagrangian approach, and test whether this, or a model of isolation by distance best fits phylogeographic patterns in coastal *Ochthebius*.

## Results

### Simulated population connectivity

**Fig. 1:**
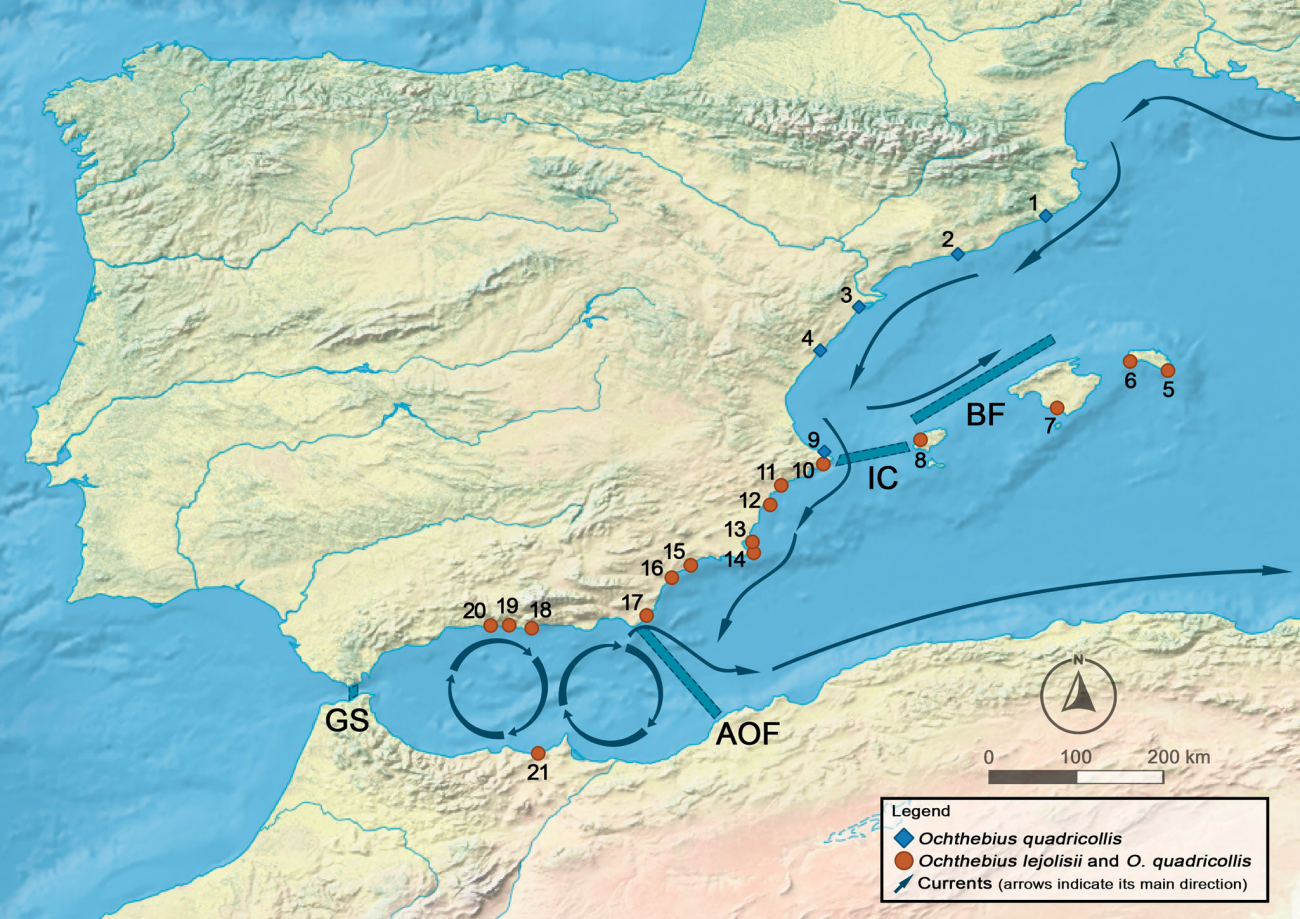
Distribution map for *Ochthebius quadricollis* and *Ochthebius lejolisii* in the western Mediterranean coast. Orange circles indicate localities in which *Ochthebius quadricollis* and *Ochthebius lejolisii* were found in sympatry whilst blue diamonds represent localities exclusively for *Ochthebius quadricollis*. Arrows indicate the main direction of the surface oceanic currents. Main marine geographic barriers are as follows: Gibraltar Strait (GS), Almeria-Oran Front (AOF), Ibiza Channel (IC) and Balearic Front (BF).

**Fig. 2:**
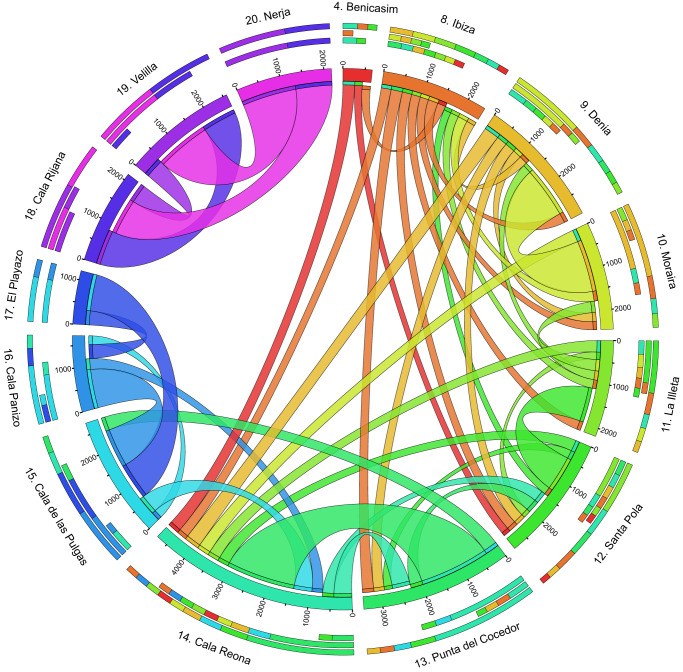
Circos diagram showing the absolute contribution of potential connectivity among the studied populations based on the biophysical model. It does not show connectivity below 0.1% of the total liberated propagules reaching another location, ignoring autoconnection. Each ribbon represents the connectivity between two populations: flow direction is indicated from source population (ribbon touching the scale) to sink population (blank space between ribbon and the scale).

To investigate the potential dispersal of *Ochthebius* by oceanic currents along the western Mediterranean coast, propagule drifting was simulated for 21 rocky shore localities (Fig. 1) using Lagrangian tools. Of 3,528,000 released propagules, the mean connection probability was 1.88% (i.e., only 66.221 propagules), of which more than half were returned to the same locality by surface currents (Fig. S1) and approximately 40% successfully arriving at other sites. Locations in Catalonia (#1 to #3: Cala Sant Francesc, Vilanova and Sant Carles de la Ràpita) and Morocco (#21: Ghansou) did not have any connection to other locations. Additional localities without an effective connectivity (bellow 0.1% of their total propagules dispersed to other localities) were in the Gymnesian Islands (i.e., Mallorca and Menorca, localities #5 to #7). Two isolated clusters were detected across remaining sites separated by the Almeria-Oran Front: populations in the Alboran Sea (#18 to #20: Cala Rijana, Velilla and Nerja) and the remaining populations (#4 and #8 to #17). The most frequent connections were detected between adjacent populations (Fig. 2), with some localities behaving as source populations (e.g., Ibiza or Denia, locations #8 and #9, respectively), others as sinks (e.g., Cala Reona - location #14, with inputs from most localities except those from the Alboran Sea and Catalonia).

Most localities showed propagule dispersal patterns from north to south, with very limited dispersal even to geographically close localities to their north. For example, propagules liberated on Ibiza disperse to the south, distant populations, such as Cala Reona, more than 200 km away, but displaying very low connectivity with a locality situated north of the Ibiza Channel. Populations located in the northern Ibiza Channel display negligible connectivity with the Balearic Islands, in most cases lower than 0.05% of realised propagules (scenario maximum of 75 propagules reaching Menorca [location #6], out of the 168.000 total propagules liberated in Vilanova i la Geltrú [location #2]). These results demonstrate the impact of the Ibiza Channel and the Balearic Front on dispersal from localities on the northern coast of the western Mediterranean.

Southern populations located close to the eastern boundary of the Almeria-Oran Front (locations #16 and #17) disperse principally from west to east, converging both west to east and north to south dispersal patterns in the southeastern Iberian Peninsula (Cala Reona - location #14).

### Isolation by distance and Mantel correlations

**Fig. 3:**
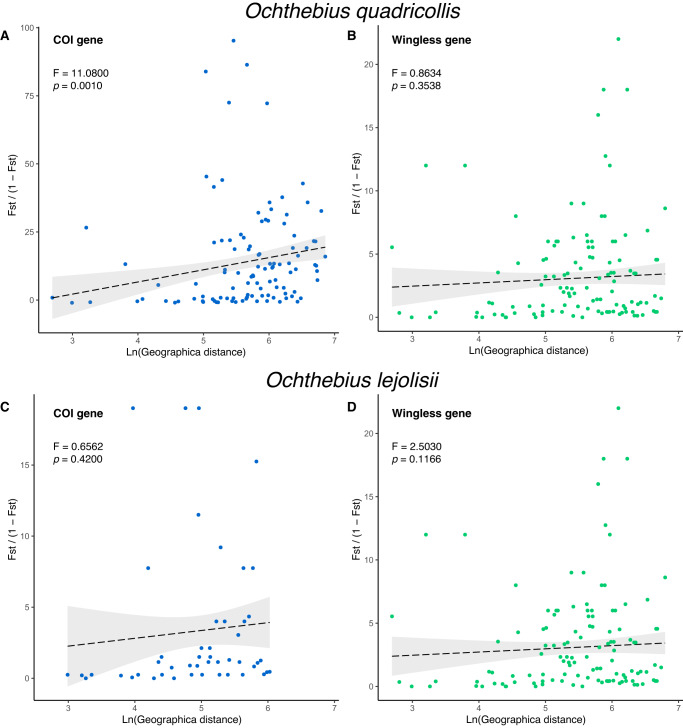
Isolation by distance plot for Euclidian distance matrices of geographic distance and linearised Fst values. *Ochthebius quadricollis* (**A,** **B**) and *Ochthebius lejolisii* (**B**, **C**) for COI (**A**, **C**) and wingless (**B**, **D**) genes.

A significant positive relationship between geographical distance and genetic differentiation was seen only for *COI* in *O. quadricollis* (Fig. 3). There was no evidence that isolation by distance had a strong influence on genetic structure for either genes in *O. lejolisii* or for *wingless* in *O. quadricollis* (Fig. 3).

**Fig. 4:**
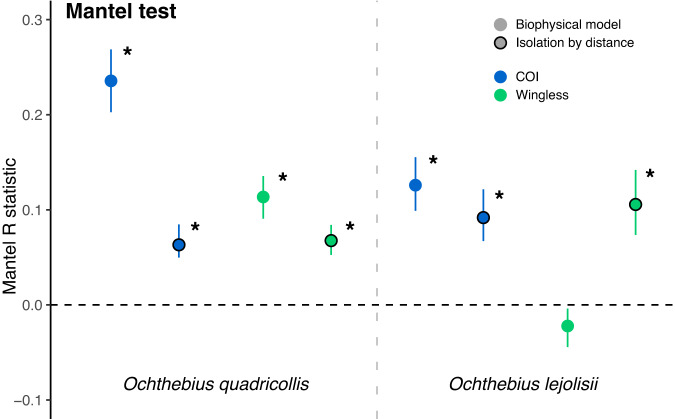
Mantel R correlations including confidence intervals of Fst pairwise values and either the biophysical model or the isolation-by-distance model. Statistical significance (assessed by two-tailed p value after 9999 permutations) is indicated by asterisks.

Mantel tests revealed that the biophysical model better explained genetic differentiation between populations than geographical distance regardless COI or wingless gene in *O. quadricollis* (Fig. 4 and Table S1). Mantel R statistics show a consistently higher correlation between the number of propagules that reach a locality (in the biophysical model) and genetic structure than with geographical distance, including 95% confidence intervals. Weaker evidence was found in O. *lejolisii*, however, where a significant correlation between biophysical model output and population differentiation was only seen in COI, with a similar Mantel R statistic confidence interval (Fig. 4 and Table S1).

## Discussion

This study explores, for the first time, whether marine currents and their associated fronts influence the genetic structure of non-marine coastal taxa by affecting population dispersal. We show that a biophysical simulation of inter-population connectivity explains the genetic structure of supratidal *Ochthebius* beetles better than the classical isolation-by-distance model, suggesting that surface water currents may play an important role in *Ochthebius*’ dispersal and connectivity/isolation. Whilst some have suggested that such marine movement may occur in coastal beetles^34^, ours is the first study to integrate population genetics with biophysical modelling to show that at least some coastal insects may primarily disperse in a manner similar to that seen in truly marine organisms. The requirement of a dry surface that allow unfolding and drying of wings during metamorphosis is clearly very rare in the ocean, and indeed being a winged insects has been suggested to be one of the primary reasons why insects are largely absent from marine systems^35^. Our results strongly suggest that although most insects, including aquatic taxa, disperse mostly through active flight^18^, rockpool beetles move between distant localities mainly in a passive manner, via ocean currents, like the majority of fully marine inhabitants of the coastal zone.

Previous studies of passive dispersal via oceanic currents have focused on long-distance dispersal by floating or rafting in brooding intertidal invertebrates lacking a planktonic/pelagic dispersal stage^36,37^, or on relatively rare events allowing terrestrial taxa to cross oceans on, for example, drifting debris^38–41^. Whilst often proposing plausible dispersal scenarios, such studies have typically been based on limited data and relied on single methodologies, our study being the first to combine population genetics with biophysical modelling. Clearly a wider application of our approach to a range of biogeographical and evolutionary scenarios may prove illuminating.

The alternative hypothesis, isolation-by-distance explaining the current genetic structure of coastal *Ochthebius*, was rejected in almost all cases due to the lack of significant relationships between genetic and geographical distance, suggesting that additional factors played a more important role in the dispersal and isolation of *Ochthebius* populations at the spatial scale studied here. Our results suggested significant isolation-by-distance only for *COI* in *O. quadricollis*, something which was not mirrored by *wingless* in this species. Despite their widespread use, mitochondrial DNA data have serious limitations for detecting isolation-by-distance, being highly susceptible to type I error (i.e., false positives) when combining distinct regional populations and not very suitable for examining fine-scale geographical patterns on their own^42^. In our study, connectivity via oceanic currents better explains present genetic structure than geographical distance in *O. quadricollis* based on Mantel tests. Interestingly, the Mantel test showed no correlation between the biophysical model and the genetic structure of the *wingless* gene of *O. lejolisii*, whereas geographical distance was apparently correlated. This may reflect the different effects that oceanic currents have on the two species. As stated above, these two beetles are broadly sympatric, but their microhabitat preferences are slightly different: *O. lejolisii* is usually found in pools further from the sea^16^, potentially meaning that oceanic currents have a lesser impact on their population connectivity than in *O. quadricollis*. These interspecific differences in habitat preference, together with differences in physiological tolerance and life cycles^20,43^ may explain the apparently different effects of oceanic currents on the two species. However, *O. lejolisii* also has a more limited distribution on the western Mediterranean coast, reaching but not crossing the Ibiza Channel. Since the effect of the Ibiza Channel is therefore not tested in our models for this species (although the effect can be seen by its absence north of the Ibiza Channel), it may be that connectivity through oceanic currents is less apparent in our statistical tests.

Of course, it is possible that other dispersal mechanisms also contribute, but likely to a lesser extent, to the genetic structure observed in these *Ochthebius*. Anemochory may contribute to the dispersal of adult *Ochthebius*, although such coastal wind mediate dispersal would be expected to act at smaller spatial scales than ocean currents. Similarly, active flight, or flight assisted by winds likely plays a limited role in the long-distance dispersal of *Ochthebius*. As observed by other authors^44^, in hypersaline inland *Ochthebius*, genetic flow amongst populations is restricted by orographic factors, even over short spatial scales in the same watershed, suggesting limited capacity for long-distance dispersal by flight. Coastal *Ochthebius* may also disperse via the transport of eggs by endo- and epizoochory with seabirds that frequently occur in rockpools. No cases of *Ochthebius* transport through avifauna have been documented, although microcrustaceans are routinely found in bird plumage^45–47^, suggesting that such mechanisms may facilitate movement of rockpool invertebrates.

Our Lagrangian model strongly suggests that the Ibiza Channel and Balearic Front act as significant dispersal barriers in *Ochthebius*, something previously suggested based on molecular data alone^7^. Our results confirm the importance of oceanographic discontinuities as barriers to connectivity and gene flow along the Mediterranean coast^9,12,48^, even for non-marine taxa. We also found that populations of *Ochthebius* in the Alboran Sea are completely isolated from other Mediterranean populations, both in Spain and Morocco. Whereas Villastrigo et al.^7^ inferred the influence of a historical barrier within the Alboran Sea^49^, simulated population connection in the biophysical model suggests that the Almeria-Oran Front and associated processes act as a significant contemporary barrier to dispersal in rockpool *Ochthebius*. The Atlantic jet flowing through the Gibraltar Strait forms two anticyclonic gyres in the Alboran Sea^50^, which may facilitate dispersal of *Ochthebius* propagules from the western to the eastern Alboran basins, and prevent connectivity in the opposite direction^24^. In addition, the Atlantic jet hinders transport between Moroccan and Iberian coasts in the Alboran Sea.

In short, our study suggests that oceanic currents may play a significant role in the dispersal of non-marine coastal taxa, despite their lack of a specific planktonic/pelagic dispersal stage^51^. By combining biophysical dispersal modelling and population genetics we show that rockpool beetles apparently disperse in a similar manner to fully marine organisms, the first time this has been demonstrated for any non-marine insect. Further work, using a wider range of molecular markers (e.g., microsatellites or SNPs), and including additional taxa, will allow us to determine whether this applies to other areas and other coastal terrestrially-derived inhabitants.

## Methods

### Study species

Our study focused on two widespread representative species of rockpool specialist *Ochthebius* lineages: *O. (Ochthebius) quadricollis* and *O. (Cobalius) lejolisii*, which are partially sympatric in the Spanish Mediterranean coast (Fig. 1)^16^. *O. quadricollis* has a wide geographical distribution in the central and western Mediterranean plus the Atlantic coast of Morocco and the Iberian Peninsula^7,16^. *O. lejolisii* is distributed principally on the Atlantic coast from the British Isles to Morocco, reaching the southeastern Iberian Mediterranean coast^16^. The study area (see below) includes two additional coastal *Ochthebius* that were not considered in this study: *Ochthebius subinteger* more common in the central Mediterranean and with a low representation in the Iberian Peninsula^16^, and *Ochthebius evae*, a poorly known flightless species that likely occupy a different microhabitat^52^.

Although species living in this fluctuating and stressful environment are expected to have similar adaptations and life history traits, some studies^21,43^ have found differences in physiological tolerances related to ancestral and recent environmental variation in their habitats, being adult and larval stages of *O. quadricollis* more tolerant to heat than *O. lejolisii*, but the latter is more resistant to salt and desiccation^52^. These different traits are likely the result of their microhabitat preference, more distant from the sea in the case of *O. lejolisii*, and nearer to the coastline, with a higher incidence of sea spray and splash, in *O. quadricollis*, for which longer hydroperiod is required^53^. Both species are multivoltine with overlapping cohorts to cope with intense and fluctuating environmental stressors (e.g., salinity, temperature, dissolved oxygen or desiccation) and highly variable water availability across the year^20^. In addition, egg hatching is more successful in *O. quadricollis* than *O. lejolisii*, which, together with its shorter overall life cycle, leads to a greater demographic success of *O. quadricollis*^20,21^. Furthermore, *Ochthebius* adults, larvae and eggs are able to survive in seawater, eggs are even resistant to extreme desiccation for several days^20,21^.

### Study area and field sampling

We focused on the western Mediterranean Sea, a complex marine system influenced by the inflow of lighter Atlantic water through the Gibraltar Strait that generates discontinuities when interacting with higher-density Mediterranean water (Fig. 1)^11^. In this area, there are several geographical barriers to the dispersal of marine organisms, including the Almeria-Oran Front, which has been suggested to play a key role in the genetic differentiation of species distributed both in the Atlantic Ocean and in the Mediterranean Sea^48,54^. Additional discontinuities that have been reported as strong barriers to gene exchange are the Balearic Front and the Ibiza Channel^12,55^.

We selected 20 rocky shore localities along the Mediterranean coast of the Iberian Peninsula and one in Morocco – from Cala Sant Francesc to Nerja plus Ghasou, representing all coastal features, including capes and gulfs. These localities were chosen non-randomly with the aim of equidistant localities but following the sites presented in Villastrigo et al.^7^ (Fig. 1). Individuals were collected from random pools following a gradient of distance from the sea in an attempt to collect both species at the localities where they are present.

### Molecular data

We used the molecular dataset from Villastrigo et al.^7^ which contains samples of both target species along the Mediterranean coast of the Iberian Peninsula, excluding samples from contiguous regions (e.g., France or the Atlantic coast of the Iberian Peninsula and Morocco). This dataset contains molecular data for three randomly selected specimens per locality for two molecular markers: 1) the 3′-end of the mitochondrial *COI* gene (829 bp) and 2) a fragment of the nuclear gene *wingless* (466 bp). Sample information including geographical data and accession numbers for DNA sequences is available in Table S2 and Fig. 1.

To reduce the molecular uncertainty caused by DNA ambiguities, we inferred haplotype sequences using the PHASE software^56^ as implemented in DNAsp 6^57^, considering 1,000 iterations, a thinning parameter of 5 and discarding the first 100 iterations as burn-in. We then calculated the fixation index (Fst) as a measure of genetic differentiation between pairs of localities using DNAsp 6^57^. Linearization of Fst values was done following the regression of Fst/(1-Fst) by Rousset^58^ in the R environment^59^ to estimate the isolation-by-distance effect.

### Biophysical model

To investigate the potential dispersal of *Ochthebius* through oceanic currents in western Mediterranean and therefore population connectivity, propagule drifting was simulated using the Lagrangian software Ichthyop v3.3^60^. This creates an individual-based, three-dimensional particle-tracking model that integrates the input time series of salinity, temperature and current velocity fields generated by the ocean model to advect and influence the virtual propagules. In this study, the term “propagule” is used to refer to resistant *Ochthebius* eggs that can be transported by oceanic currents without affecting their survival (the low floating capability of adults and larvae and the aerial respiration of adults makes them less likely to disperse via oceanic currents).

A multi-year simulation of the Western Mediterranean OPerational modelling system (WMOP)^61,62^, developed by the Balearic Islands Coastal Observing and Forecasting System^63^ was used to simulate propagule trajectories and velocity. WMOP is based on the regional configuration of the Regional Oceanic Modeling System ROMS model^64^ implemented over the western Mediterranean Sea, which is a free-surface, split-explicit model solving primitive hydrostatic equations using terrain-following curvilinear vertical coordinates, with a horizontal resolution of ca. 2 km and 32 sigma-levels in the vertical dimension. It covers an area from the Gibraltar Strait to the Sardinia Channel (6°W to 9.2°E and 35°N to 44.5°N). The model is forced by high-resolution atmospheric forcing (5 km, 3 h) from the High-Resolution Limited Area Model (HIRLAM) simulations developed by the Spanish Meteorological Agency. The simulation used in this study is a free-run hindcast simulation of WMOP, spanning the period 2009-2015. Initial and boundary conditions were provided by the CMEMS Mediterranean model reanalysis^65^. Further details, evaluation and validation of this high-resolution WMOP simulation were presented by Mourre et al.^62^ and Aguiar et al.^66^. In particular, the simulation was shown to accurately represent the main circulation features (including the Atlantic Jet, Alboran gyres and the Algerian, Northern and Balearic Currents), as well as the spatial variability of surface eddy kinetic energy.

The dispersal model considered the release of 1000 particles representing virtual *Ochthebius* propagules from each study site. For this purpose, the release zone is defined by the geographical coordinates of a 50 metres radius polygon centred 100 m offshore and extending from 0 to 1 m depth. Particle releases were scheduled every 15 days from January to December throughout the study period (2009-2015) to cover several consecutive reproductive seasons and record the annual and interannual variability in marine currents. In total, 3,528,000 propagules were released (1000 propagules × 7 years × 12 months × 2 releases per month × 21 localities). After release, the location of each propagule was tracked for 5 days considering the at-sea duration of viable propagules, based on the expected hatching time^20^. This model was chosen to produce the most realistic but conservative dispersal and connectivity scenario, whilst keeping the computational cost to a manageable level.

Ichthyop output files gathered all possible propagule locations at the end of the marine dispersal period, which were analysed using geographic information systems. Twenty-one rockpool recruitment areas were then considered, from Cala Sant Francesc in the north (Catalonia, Spain) to Ghansou (Morocco) in the south. Virtual propagules were considered to have reached a recruitment area when they reached a minimum buffer distance of approximately 10 km radius from the epicentre of any other studied rockpool locality. This buffer size was considered to cover a reasonable water area surrounding the site, but to avoid overlapping the buffer zones of closely spaced sites. Raw values of pairwise potential connectivity were calculated and visualised using Circos^67^. The spread of propagules throughout our biophysical model does not take into account their survival during the dispersal event (e.g., predation by other organisms) and/or their establishment on new rocky shores. For this purpose, we considered two localities to be positively connected when the number of propagules reaching a locality was equal to or greater than 0.1% of the initial number of propagules dispersed from the source locality.

### Data analyses

The relationship between genetic differentiation and geographical distance between studied localities was explored using isolation by distance (IBD) analysis. A linear model (*lm* function of the R-package *stats*^59^) was used to fit linearised Fst values against the natural logarithm of geographical distance between populations. Linear geographical distances between pairs of locations were computed using the vector analysis tool in QGIS 3.16.13.

To assess whether the simulated oceanic connection or IBD best fits observed genetic patterns, Euclidean distance matrices for the following variables were generated using the *dist* function of the R-package *stats*^59^: Fst values, geographical distance and the number of simulated propagules dispersed between each pair of localities (raw data available in Table S3). Due to the lack of genetic data for multiple specimens, we discarded six localities (numbers 2, 4, 6, 7, 8 and 21) in subsequent analyses. Mantel tests were calculated for the Fst distance matrix against the distance matrices of the other variables using the Spearman correlation method under the *mantel* function of the R-package *ecodist*^68^ with 9,999 permutations. Bootstrapped confidence intervals (95%) for the Mantel test were calculated using 9999 iterations.

### Reporting summary

Further information on research design is available in the Nature Research Reporting Summary linked to this article.

### Supplementary information

Supplementary Information

Reporting Summary

## Data Availability

The data is fully available in the Supplementary material.
