# Supplementary material for: Oceanic currents maintain the genetic structure of non-marine coastal taxa in the western Mediterranean Sea
Source: NPJ Biodivers. 2023 Nov 20;2:25. doi: 10.1038/s44185-023-00028-0 (PMC11332052; doi:10.1038/s44185-023-00028-0)
Supplement: Supplementary file 1 — Supplementary Information [file 44185_2023_28_MOESM1_ESM.pdf]

Supplementary Information for

Oceanic currents maintain the genetic structure of non-marine coastal taxa in the western Mediterranean Sea

Adrián Villastrigo[1†*], Víctor Orenes-Salazar[2†], Antonio José García-Meseguer[2], Juana María Mirón-Gatón[2], Baptiste Mourre[3], Andrés Millán[2], Josefa Velasco[2]

1: Division of Entomology, SNSB-Zoologische Staatssammlung München, Münchhausenstraße 21, 81247 München, Germany
2: Departamento de Ecología e Hidrología, Universidad de Murcia, 30100 Murcia, Spain
3: Balearic Islands Coastal Observing and Forecasting System, 07121 Palma, Spain
†These authors contributed equally to this work

*Corresponding author: adrianvillastrigo@um.es

This file includes:

Figure S1. Circos diagram showing the absolute contribution of potential connectivity among the studied populations based on the biophysical model. It does not show connectivity below 0.1% of the total liberated propagules reaching another location, considering autoconnection and a shared scale. Each ribbon represent the connectivity between two populations: flow direction is indicated from source population (ribbon touching the scale) to sink population (blank space between ribbon and the scale).

Table S1. Mantel tests statistics (R, confidence interval and two-tailed p value) for the correlation of genetic distance with both propagule connectivity and geographic distance. Bold values indicate analyses with significant p value.

Table S2. List of samples per locality with information regarding geographical location, voucher, and accession numbers.

Table S3. Raw data for pairs of localities used for statistical analyses, including geographic distance, propagules connected by the biophysical model, and Fst for each loci.

Figure S1. Circos diagram showing the absolute contribution of potential connectivity among the studied populations based on the biophysical model. It does not show connectivity below 0.1% of the total liberated propagules reaching another location, considering autoconnection and a shared scale. Each ribbon represent the connectivity between two populations: flow direction is indicated from source population (ribbon touching the scale) to sink population (blank space between ribbon and the scale).

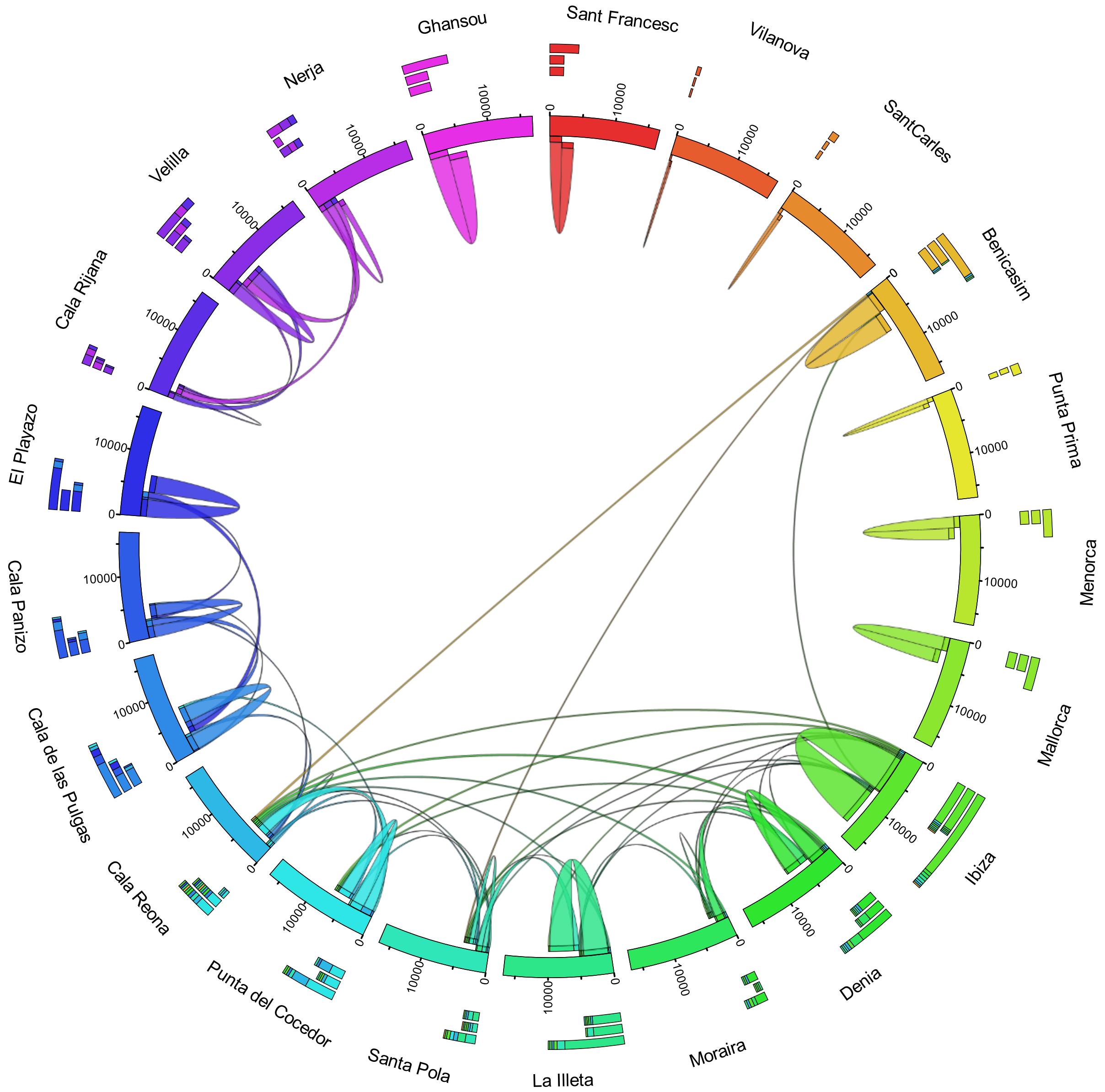

Table S1. Mantel tests statistics (R, confidence interval and two-tailed *p* value) for the correlation of genetic distance with both propagule connectivity and geographic distance. Bold values indicate analyses with significant *p* value.

Species	Gene	Statistic	Marine current connectivity (nº propagules)	Geographic distance
O. quadricollis	COI	Mantel R	**0.2356 (0.2027 – 0.2687)**	**0.0632 (0.0498 – 0.0846)**
		p value	**0.0001**	**0.0091**
	Wingless	Mantel R	**0.1135 (0.0907 – 0.1355)**	**0.0676 (0.0526 – 0.0841)**
		p value	**0.0001**	**0.0009**
O. lejolisii	COI	Mantel R	**0.1259 (0.0990 – 0.1553)**	**0.0918 (0.0671 – 0.1215)**
		p value	**0.0001**	**0.0001**
	Wingless	Mantel R	-0.0222 (-0.0443 – -0.0039)	**0.1056 (0.0736 – 0.1419)**
		p value	0.5104	**0.0004**

**Table S2**. List of samples per locality with information regarding its geographical location, voucher and accession numbers						
**Locality ID**	**Locality name**	**GPS**	**Species**	**Sample Voucher**	**COI**	**Wingless**
1	Cala Sant Francesc, Girona, Spain	41.679152, 2.807989	*O. quadricollis*	AV217	OU595503.1	OU595642.1
				AV268	OU595504.1	OU595643.1
				AV269	OU595505.1	OU595644.1
2	Vilanova I la Geltrú, Barcelona, Spain	41.218230, 1.748436	-	-	-	-
3	Sant Carles de la Ràpita, Tarragona, Spain	40.580735, 0.554946	*O. quadricollis*	AV242	OU595562.1	OU595684.1
				AV272	OU595563.1	OU595685.1
				AV273	OU595564.1	OU595686.1
4	Benicàssim, Castellón, Spain	40.056089, 0.088872	-	-	-	-
5	Punta Prima, Menorca, Spain	39.812500, 4.280611	*O. quadricollis*	AV367	OU595558.1	OU595682.1
				AV376	OU595559.1	-
				AV377	OU595560.1	OU595683.1
6	Cap d'Artrutx, Menorca, Spain	39.923194, 3.824638	-	-	-	-
7	Sa Rapita, Mallorca, Spain	39.362417, 2.945000	-	-	-	-
8	Sant Antoni de Portmany, Ibiza	38.978639, 1.298250	-	-	-	-
9	Denia, Alicante, Spain	38.833528, 0.138467	*O. quadricollis*	AV238	OU595519.1	OU595654.1
				AV276	OU595520.1	OU595655.1
				AV277	OU595521.1	OU595656.1
10	Moraira, Alicante, Spain	38.685178, 0.125989	*O. quadricollis*	AV236	OU595539.1	OU595668.1
				AV280	OU595540.1	OU595669.1
				AV281	OU595541.1	OU595670.1
			O. lejolisii	AV237	OU595447.1	OU595602.1
				AV278	OU595448.1	OU595603.1
				AV279	OU595449.1	OU595604.1
11	La illeta, Alicante, Spain	38.431458, -0.380934	*O. quadricollis*	AV234	OU595533.1	OU595664.1
				AV284	OU595534.1	OU595665.1
				AV285	OU595535.1	OU595666.1
			O. lejolisii	AV235	OU595442.1	OU595599.1
				AV282	OU595443.1	OU595600.1
				AV283	OU595444.1	OU595601.1
12	Cabo de Santa Pola, Alicante, Spain	38.197111, -0.514417	*O. quadricollis*	AV232	OU595494.1	OU595633.1
				AV288	OU595495.1	OU595634.1
				AV289	OU595496.1	OU595635.1
			O. lejolisii	AV233	OU595414.1	OU595572.1
				AV286	OU595415.1	OU595573.1
				AV287	OU595416.1	OU595574.1
13	Punta del Cocedor, Murcia, Spain	37.749964, -0.728281	*O. quadricollis*	AV363	OU595555.1	OU595679.1
				AV364	OU595556.1	OU595680.1
				AV365	OU595557.1	OU595681.1
			O. lejolisii	AV290	OU595459.1	OU595612.1
				AV291	OU595460.1	OU595613.1
				AV292	OU595461.1	OU595614.1
14	Cala Reona, Murcia, Spain	37.617328, -0.712847	*O. quadricollis*	AV296	OU595510.1	OU595648.1
				AV297	OU595511.1	OU595649.1
				AV298	OU595512.1	OU595650.1
			O. lejolisii	AV299	OU595426.1	OU595584.1
				AV300	OU595427.1	OU595585.1
				AV301	OU595428.1	OU595586.1
15	Cala de las Pulgas, Murcia, Spain	37.467717, -1.471239	*O. quadricollis*	AV353	OU595500.1	OU595639.1
				AV354	OU595501.1	OU595640.1
				AV355	OU595502.1	OU595641.1
			O. lejolisii	AV347	OU595420.1	OU595578.1
				AV348	OU595421.1	OU595579.1
				AV349	OU595422.1	OU595580.1
16	Cala Panizo, Almería, Spain	37.318997, -1.700153	*O. quadricollis*	AV248	OU595552.1	-
				AV309	OU595553.1	OU595677.1
				AV310	OU595554.1	OU595678.1
			O. lejolisii	AV249	OU595457.1	-
				AV308	OU595458.1	OU595611.1
17	El Playazo, Almería, Spain	36.862972, -2.003934	*O. quadricollis*	AV320	OU595522.1	OU595657.1
				AV321	OU595523.1	OU595658.1
				AV322	OU595524.1	OU595659.1
			O. lejolisii	AV317	OU595435.1	OU595592.1
				AV318	OU595436.1	OU595593.1
				AV319	OU595437.1	OU595594.1
18	Cala Rijana, Granada, Spain	36.709143, -3.389723	*O. quadricollis*	AV381	OU595513.1	OU595651.1
				AV382	OU595514.1	OU595652.1
				AV383	OU595515.1	OU595653.1
			O. lejolisii	AV386	OU595429.1	OU595587.1
				AV387	OU595430.1	OU595588.1
				AV388	OU595431.1	OU595589.1
19	Velilla, Granada, Spain	36.744903, -3.661633	*O. quadricollis*	AV326	OU595567.1	OU595688.1
				AV327	OU595568.1	OU595689.1
				AV328	OU595569.1	OU595690.1
			O. lejolisii	AV323	OU595467.1	OU595616.1
				AV324	OU595468.1	OU595617.1
				AV325	OU595469.1	OU595618.1
20	Nerja, Málaga, Spain	36.740919, -3.884114	*O. quadricollis*	AV332	OU595542.1	OU595671.1
				AV333	OU595543.1	OU595672.1
				AV334	OU595544.1	OU595673.1
			O. lejolisii	AV329	OU595450.1	OU595605.1
				AV330	OU595451.1	OU595606.1
				AV331	OU595452.1	OU595607.1
21	Ghansou beach, Boudiar, Morocco	35.199000, -3.311028	-	-	-	-

Table S3. Raw data for pairs of localities used for statistical analyses, including geographic distance, propagules connected by the biophysical model, and Fst for each loci.

id	Source Locality	Source ID	Sink locality	Sink ID	Geographic distance (metres)	Propagules (raw value)	Propagules (effective)	Fst value COI (*quadricollis*)	Fst value Wingless (*quadricollis*)	Fst value COI (*lejolisii*)	Fst value Wingless (*lejolsii*)
1	Santa Pola	12	Santa Pola	12	0	667	667	NA	NA	NA	NA
2	Santa Pola	12	La illeta	11	28512,5	831	831	0,46667	0,28462	0,2	0,28571
3	Santa Pola	12	Denia	9	90736,1	67	0	0,175	0,45294	NA	NA
4	Santa Pola	12	Moraira	10	77852,9	58	0	0,38065	0,16	0,53333	0,28571
5	Santa Pola	12	Benicasim	4	212869,4	21	0	NA	NA	NA	NA
6	Santa Pola	12	Cala Reona	14	66676,9	267	267	0,5651	0,52308	0,88571	0
7	Santa Pola	12	Cala de las Pulgas	15	116827,6	51	0	0,53333	0,46667	0,95	0,05714
8	Santa Pola	12	Punta del Cocedor	13	53069,6	197	197	0,36471	0,27143	0,95	0,28571
9	Santa Pola	12	Sant Carles de la Ràpita	3	280207,2	3	0	0,87797	0,40741	NA	NA
10	Santa Pola	12	Cala Panizo	16	142884,6	28	0	0,2396	0,48571	0,95	0
11	Santa Pola	12	El Playazo	17	198133,8	21	0	0,92329	0,49	0,90204	0,04545
12	Santa Pola	12	Cala Sant Francesc	1	479613,8	1	0	0,84561	0,47416	NA	NA
13	Santa Pola	12	Vilanova i la Geltrú	2	387483,2	1	0	NA	NA	NA	NA
14	Santa Pola	12	Cala Rijana	18	303284,3	4	0	0,90649	0,56667	0,8129	0,29091
15	Santa Pola	12	Velilla	19	321661,7	1	0	0,87771	0,58667	0,88571	0,36923
16	Santa Pola	12	Ibiza	8	18018,6	176	176	0,37143	NA	NA	NA
17	Santa Pola	12	Nerja	20	339050,5	4	0	0,83912	0,58667	0,93846	0,02857
18	Santa Pola	12	Cap d'Artrutx	6	421550,9	22	0	NA	NA	NA	NA
19	Santa Pola	12	Punta Prima	5	452327,9	23	0	0,28	0,2	NA	NA
20	Santa Pola	12	Mallorca	7	327226,5	33	0	NA	NA	NA	NA
21	Santa Pola	12	Ghansou	21	416061,4	1	0	0,89231	0,74	NA	NA
22	La illeta	11	Santa Pola	12	28512,5	180	180	0,46667	0,28462	0,2	0,28571
23	La illeta	11	La illeta	11	0	3881	3881	NA	NA	NA	NA
24	La illeta	11	Denia	9	63540,9	217	217	0,77778	0,53846	NA	NA
25	La illeta	11	Moraira	10	52397,7	266	266	0,92381	0,03077	0,05714	0
26	La illeta	11	Benicasim	4	184869,6	79	0	NA	NA	NA	NA
27	La illeta	11	Cala Reona	14	94948,8	273	273	0,68302	0,88889	0,42857	0
28	La illeta	11	Cala de las Pulgas	15	143615,4	20	0	0,94286	0,81905	0,6	0,05714
29	La illeta	11	Punta del Cocedor	13	81550,8	159	0	0,4	0	0,6	0,28571
30	La illeta	11	Sant Carles de la Ràpita	3	251828,9	8	0	0,96129	0,79459	NA	NA
31	La illeta	11	Cala Panizo	16	169459,3	24	0	0,40571	0,85	0,6	0
32	La illeta	11	El Playazo	17	22542,7	16	0	0,98961	0,86316	0,56364	0,04545
33	La illeta	11	Cala Sant Francesc	1	451669,4	0	0	0,93333	0,81849	NA	NA
34	La illeta	11	Vilanova i la Geltrú	2	359097,7	3	0	NA	NA	NA	NA
35	La illeta	11	Cala Rijana	18	327371,1	4	0	0,97037	0,94118	0,45373	0,29091
36	La illeta	11	Velilla	19	34492,4	1	0	0,96735	0,88889	0,52	0,36923
37	La illeta	11	Ibiza	8	158187,1	205	205	0,9027	NA	NA	NA
38	La illeta	11	Nerja	20	361817,4	2	0	0,92525	0,88889	0,55556	0,02857
39	La illeta	11	Cap d'Artrutx	6	399302,3	23	0	NA	NA	NA	NA
40	La illeta	11	Punta Prima	5	431222,1	14	0	0,85	0,5	NA	NA
41	La illeta	11	Mallorca	7	306458,2	29	0	NA	NA	NA	NA
42	La illeta	11	Ghansou	21	443821,9	2	0	0,984	0,95652	NA	NA
43	Denia	9	Santa Pola	12	90736,1	244	244	0,175	0,45294	NA	NA
44	Denia	9	La illeta	11	63540,9	164	0	0,77778	0,53846	NA	NA
45	Denia	9	Denia	9	0	2427	2427	NA	NA	NA	NA
46	Denia	9	Moraira	10	16504,1	212	212	0,2	0,25882	NA	NA
47	Denia	9	Benicasim	4	135800,7	140	0	NA	NA	NA	NA
48	Denia	9	Cala Reona	14	154210,5	364	364	0,57956	0,85714	NA	NA
49	Denia	9	Cala de las Pulgas	15	20709,5	75	0	0,6	0,77647	NA	NA
50	Denia	9	Punta del Cocedor	13	142182,3	253	253	0,7	0,29091	NA	NA
51	Denia	9	Sant Carles de la Ràpita	3	197250,4	108	0	0,91724	0,7697	NA	NA
52	Denia	9	Cala Panizo	16	232982,6	77	0	0,23516	0,8125	NA	NA
53	Denia	9	El Playazo	17	288758,2	42	0	0,95362	0,82667	NA	NA
54	Denia	9	Cala Sant Francesc	1	389077,4	51	0	0,88571	0,81217	NA	NA
55	Denia	9	Vilanova i la Geltrú	2	298310,8	75	0	NA	NA	NA	NA
56	Denia	9	Cala Rijana	18	390104,9	26	0	0,93425	0,92308	NA	NA
57	Denia	9	Velilla	19	40710,2	24	0	0,92899	0,85714	NA	NA
58	Denia	9	Ibiza	8	101881,4	206	206	0,35385	NA	NA	NA
59	Denia	9	Nerja	20	423602,2	28	0	0,89267	0,85714	NA	NA
60	Denia	9	Cap d'Artrutx	6	33983,8	24	0	NA	NA	NA	NA
61	Denia	9	Punta Prima	5	373309,6	17	0	0,4	0,3	NA	NA
62	Denia	9	Mallorca	7	249771,5	47	0	NA	NA	NA	NA
63	Denia	9	Ghansou	21	506788,2	26	0	0,94286	0,94737	NA	NA
64	Moraira	10	Santa Pola	12	77852,9	134	0	0,38065	0,16	0,53333	0,28571
65	Moraira	10	La illeta	11	52397,7	54	0	0,92381	0,03077	0,05714	0
66	Moraira	10	Denia	9	16504,1	1235	1235	0,2	0,25882	NA	NA
67	Moraira	10	Moraira	10	0	227	227	NA	NA	NA	NA
68	Moraira	10	Benicasim	4	152235,7	46	0	NA	NA	NA	NA
69	Moraira	10	Cala Reona	14	139481,3	293	293	0,65	0,72	0,46667	0,2
70	Moraira	10	Cala de las Pulgas	15	194676,8	30	0	0,93846	0,66667	0,2	0,6
71	Moraira	10	Punta del Cocedor	13	127955,4	137	0	0,87368	0	0,2	0
72	Moraira	10	Sant Carles de la Ràpita	3	213655,8	15	0	0,95789	0,625	NA	NA
73	Moraira	10	Cala Panizo	16	220716,5	35	0	0,38824	0,69412	0,2	0
74	Moraira	10	El Playazo	17	27587,6	23	0	0,98857	0,7	0,2	0
75	Moraira	10	Cala Sant Francesc	1	403302,4	1	0	0,92727	0,6717	NA	NA
76	Moraira	10	Vilanova i la Geltrú	2	313553,8	5	0	NA	NA	NA	NA
77	Moraira	10	Cala Rijana	18	379737,8	2	0	0,96757	0,77143	0,21887	0,3
78	Moraira	10	Velilla	19	397317,1	1	0	0,97143	0,77895	0,3	0,46667
79	Moraira	10	Ibiza	8	106873,8	236	236	0,67273	NA	NA	NA
80	Moraira	10	Nerja	20	414170,9	2	0	0,93451	0,77895	0,31429	0,4
81	Moraira	10	Cap d'Artrutx	6	347335,3	22	0	NA	NA	NA	NA
82	Moraira	10	Punta Prima	5	379800,8	13	0	0,73333	0,29231	NA	NA
83	Moraira	10	Mallorca	7	255421,1	27	0	NA	NA	NA	NA
84	Moraira	10	Ghansou	21	493284,1	0	0	0,98545	0,86667	NA	NA
85	Benicasim	4	Santa Pola	12	212869,4	178	178	NA	NA	NA	NA
86	Benicasim	4	La illeta	11	184869,6	73	0	NA	NA	NA	NA
87	Benicasim	4	Denia	9	135800,7	94	0	NA	NA	NA	NA
88	Benicasim	4	Moraira	10	152235,7	132	0	NA	NA	NA	NA
89	Benicasim	4	Cala Reona	14	279534,4	281	281	NA	NA	NA	NA
90	Benicasim	4	Cala de las Pulgas	15	31771,1	9	0	NA	NA	NA	NA
91	Benicasim	4	Punta del Cocedor	13	265639,4	123	0	NA	NA	NA	NA
92	Benicasim	4	Sant Carles de la Ràpita	3	70449,6	23	0	NA	NA	NA	NA
93	Benicasim	4	Cala Panizo	16	341372,8	2	0	NA	NA	NA	NA
94	Benicasim	4	El Playazo	17	398718,2	12	0	NA	NA	NA	NA

Continue to the next page

Continue from the previous page

id	Source Locality	Source ID	Sink locality	Sink ID	Geographic distance (metres)	Propagules (raw value)	Propagules (effective)	Fst value COI (*quadricollis*)	Fst value Wingless (*quadricollis*)	Fst value COI (*lejolisii*)	Fst value Wingless (*lejolsii*)
95	Benicasim	4	Cala Sant Francesc	1	291579,6	0	0	NA	NA	NA	NA
96	Benicasim	4	Vilanova i la Geltrú	2	190686,2	2	0	NA	NA	NA	NA
97	Benicasim	4	Benicasim	4	0	3157	3157	NA	NA	NA	NA
98	Benicasim	4	Cala Rijana	18	47991,9	1	0	NA	NA	NA	NA
99	Benicasim	4	Velilla	19	492268,7	0	0	NA	NA	NA	NA
100	Benicasim	4	Ibiza	8	158506,1	126	0	NA	NA	NA	NA
101	Benicasim	4	Nerja	20	505724,6	0	0	NA	NA	NA	NA
102	Benicasim	4	Cap d'Artrutx	6	319377,2	21	0	NA	NA	NA	NA
103	Benicasim	4	Punta Prima	5	359277,3	13	0	NA	NA	NA	NA
104	Benicasim	4	Mallorca	7	256736,6	36	0	NA	NA	NA	NA
105	Benicasim	4	Ghansou	21	616870,6	0	0	NA	NA	NA	NA
106	Cala Reona	14	Santa Pola	12	66676,9	269	269	0,5651	0,52308	0,88571	0
107	Cala Reona	14	La illeta	11	94948,8	95	0	0,68302	0,88889	0,42857	0,2
108	Cala Reona	14	Denia	9	154210,5	69	0	0,57956	0,85714	NA	NA
109	Cala Reona	14	Moraira	10	139481,3	49	0	0,65	0,72	0,46667	0,2
110	Cala Reona	14	Benicasim	4	279534,4	12	0	NA	NA	NA	NA
111	Cala Reona	14	Cala Reona	14	0	293	293	NA	NA	NA	NA
112	Cala Reona	14	Cala de las Pulgas	15	69049,2	86	0	0,62791	0,18571	1	0,12727
113	Cala Reona	14	Punta del Cocedor	13	14784,2	427	427	0,6549	0,84706	1	0,2
114	Cala Reona	14	Sant Carles de la Ràpita	3	346776,4	4	0	0,72063	0,24	NA	NA
115	Cala Reona	14	Cala Panizo	16	93404,9	24	0	0,08475	0,25	1	0
116	Cala Reona	14	El Playazo	17	141887,9	38	0	0,38462	0,13333	0,92	0
117	Cala Reona	14	Cala Sant Francesc	1	542745,1	0	0	0,70462	0,55	NA	NA
118	Cala Reona	14	Vilanova i la Geltrú	2	452442,5	1	0	NA	NA	NA	NA
119	Cala Reona	14	Cala Rijana	18	258230,6	6	0	0,41333	0	0,75294	0,24
120	Cala Reona	14	Velilla	19	279163,4	6	0	0,74545	0,85714	0,88571	0,33333
121	Cala Reona	14	Ibiza	8	231898,8	102	0	0,65657	NA	NA	NA
122	Cala Reona	14	Nerja	20	297920,3	12	0	0,71579	0,85714	1	0,05714
123	Cala Reona	14	Cap d'Artrutx	6	470031,7	24	0	NA	NA	NA	NA
124	Cala Reona	14	Punta Prima	5	497876,9	13	0	0,62553	0,3	NA	NA
125	Cala Reona	14	Mallorca	7	373272,6	26	0	NA	NA	NA	NA
126	Cala Reona	14	Ghansou	21	355388,2	3	0	0,76119	0,94737	NA	NA
127	Cala de las Pulgas	15	Santa Pola	12	116827,6	39	0	0,53333	0,46667	0,95	0,05714
128	Cala de las Pulgas	15	La illeta	11	143615,4	9	0	0,94286	0,81905	0,6	0,6
129	Cala de las Pulgas	15	Denia	9	20709,5	13	0	0,6	0,77647	NA	NA
130	Cala de las Pulgas	15	Moraira	10	194676,8	27	0	0,93846	0,66667	0,2	0,6
131	Cala de las Pulgas	15	Benicasim	4	31771,1	11	0	NA	NA	NA	NA
132	Cala de las Pulgas	15	Cala Reona	14	69049,2	438	438	0,62791	0,18571	1	0,12727
133	Cala de las Pulgas	15	Cala de las Pulgas	15	0	2383	2383	NA	NA	NA	NA
134	Cala de las Pulgas	15	Punta del Cocedor	13	72696,4	105	0	0,86667	0,78	0	0,6
135	Cala de las Pulgas	15	Sant Carles de la Ràpita	3	387554,7	2	0	0,97333	0,3	NA	NA
136	Cala de las Pulgas	15	Cala Panizo	16	26140,6	207	207	0,2	0	0	0
137	Cala de las Pulgas	15	El Playazo	17	82115,2	35	0	1	0	0,2	0,22857
138	Cala de las Pulgas	15	Cala Sant Francesc	1	594652,6	0	0	0,94483	0,50909	NA	NA
139	Cala de las Pulgas	15	Vilanova i la Geltrú	2	500327,3	0	0	NA	NA	NA	NA
140	Cala de las Pulgas	15	Cala Rijana	18	19020,8	8	0	0,97808	0,3	0,53333	0,45
141	Cala de las Pulgas	15	Velilla	19	210569,4	10	0	0,98644	0,65455	0,8	0,52
142	Cala de las Pulgas	15	Ibiza	8	294831,7	71	0	0,90476	NA	NA	NA
143	Cala de las Pulgas	15	Nerja	20	229136,7	28	0	0,95126	0,65455	1	0
144	Cala de las Pulgas	15	Cap d'Artrutx	6	535174,9	14	0	NA	NA	NA	NA
145	Cala de las Pulgas	15	Punta Prima	5	564219,9	18	0	0,8	0,12	NA	NA
146	Cala de las Pulgas	15	Mallorca	7	439233,1	21	0	NA	NA	NA	NA
147	Cala de las Pulgas	15	Ghansou	21	301084,8	3	0	1	0,825	NA	NA
148	Punta del Cocedor	13	Santa Pola	12	53069,6	289	289	0,36471	0,27143	0,95	0,28571
149	Punta del Cocedor	13	La illeta	11	81550,8	59	0	0,4	0	0,6	0
150	Punta del Cocedor	13	Denia	9	142182,3	19	0	0,7	0,29091	NA	NA
151	Punta del Cocedor	13	Moraira	10	127955,4	26	0	0,87368	0	0,2	0
152	Punta del Cocedor	13	Benicasim	4	265639,4	13	0	NA	NA	NA	NA
153	Punta del Cocedor	13	Cala Reona	14	14784,2	1661	1661	0,6549	0,84706	1	0,2
154	Punta del Cocedor	13	Cala de las Pulgas	15	72696,4	328	328	0,86667	0,78	0	0,6
155	Punta del Cocedor	13	Punta del Cocedor	13	0	1923	1923	NA	NA	NA	NA
156	Punta del Cocedor	13	Sant Carles de la Ràpita	3	333251,1	2	0	0,94667	0,75556	NA	NA
157	Punta del Cocedor	13	Cala Panizo	16	98317,5	29	0	0,34545	0,81053	0	0
158	Punta del Cocedor	13	El Playazo	17	14993,1	23	0	0,97867	0,82222	0,2	0
159	Punta del Cocedor	13	Cala Sant Francesc	1	531171,8	0	0	0,91724	0,78644	NA	NA
160	Punta del Cocedor	13	Vilanova i la Geltrú	2	440036,1	0	0	NA	NA	NA	NA
161	Punta del Cocedor	13	Cala Rijana	18	262904,4	14	0	0,95949	0,9	0,53333	0,3
162	Punta del Cocedor	13	Velilla	19	283132,6	19	0	0,95102	0,84706	0,8	0,46667
163	Punta del Cocedor	13	Ibiza	8	223528,6	121	0	0,84242	NA	NA	NA
164	Punta del Cocedor	13	Nerja	20	301543,3	22	0	0,90909	0,84706	1	0,4
165	Punta del Cocedor	13	Cap d'Artrutx	6	463017,9	26	0	NA	NA	NA	NA
166	Punta del Cocedor	13	Punta Prima	5	491677,9	17	0	0,77143	0,41538	NA	NA
167	Punta del Cocedor	13	Mallorca	7	366771,7	30	0	NA	NA	NA	NA
168	Punta del Cocedor	13	Ghansou	21	365625,3	8	0	0,968	0,92727	NA	NA
169	Sant Carles de la Ràpita	3	Santa Pola	12	280207,2	124	0	0,87797	0,40741	NA	NA
170	Sant Carles de la Ràpita	3	La illeta	11	251828,9	45	0	0,96129	0,79459	NA	NA
171	Sant Carles de la Ràpita	3	Denia	9	197250,4	45	0	0,91724	0,7697	NA	NA
172	Sant Carles de la Ràpita	3	Moraira	10	213655,8	39	0	0,95789	0,625	NA	NA
173	Sant Carles de la Ràpita	3	Benicasim	4	70449,6	8	0	NA	NA	NA	NA
174	Sant Carles de la Ràpita	3	Cala Reona	14	346776,4	145	0	0,72063	0,24	NA	NA
175	Sant Carles de la Ràpita	3	Cala de las Pulgas	15	387554,7	12	0	0,97333	0,3	NA	NA
176	Sant Carles de la Ràpita	3	Punta del Cocedor	13	333251,1	71	0	0,94667	0,75556	NA	NA
177	Sant Carles de la Ràpita	3	Sant Carles de la Ràpita	3	0	479	479	NA	NA	NA	NA
178	Sant Carles de la Ràpita	3	Cala Panizo	16	41146,1	2	0	0,65161	0,31429	NA	NA
179	Sant Carles de la Ràpita	3	El Playazo	17	468823,7	9	0	0,9746	0,26667	NA	NA
180	Sant Carles de la Ràpita	3	Cala Sant Francesc	1	225093,1	0	0	0,63077	0,1	NA	NA
181	Sant Carles de la Ràpita	3	Vilanova i la Geltrú	2	122984,3	70	0	NA	NA	NA	NA
182	Sant Carles de la Ràpita	3	Cala Rijana	18	549995,7	0	0	0,95224	0,3	NA	NA
183	Sant Carles de la Ràpita	3	Velilla	19	561987,7	1	0	0,96066	0,77647	NA	NA
184	Sant Carles de la Ràpita	3	Ibiza	8	188933,1	117	0	0,95772	NA	NA	NA
185	Sant Carles de la Ràpita	3	Nerja	20	575135,9	1	0	0,92437	0,77647	NA	NA
186	Sant Carles de la Ràpita	3	Cap d'Artrutx	6	287584,8	28	0	NA	NA	NA	NA
187	Sant Carles de la Ràpita	3	Punta Prima	5	328473,6	22	0	0,94576	0,32308	NA	NA
188	Sant Carles de la Ràpita	3	Mallorca	7	244911,4	21	0	NA	NA	NA	NA

Continue to the next page

Continue from the previous page

id	Source Locality	Source ID	Sink locality	Sink ID	Geographic distance (metres)	Propagules (raw value)	Propagules (effective)	Fst value COI (*quadricollis*)	Fst value Wingless (*quadricollis*)	Fst value COI (*lejolisii*)	Fst value Wingless (*lejolsii*)
189	Sant Carles de la Ràpita	3	Ghansou	21	687173,3	0	0	0,97333	0,87273	NA	NA
190	Cala Panizo	16	Santa Pola	12	142884,6	36	0	0,2396	0,48571	0,95	0
191	Cala Panizo	16	La illeta	11	169459,3	3	0	0,40571	0,85	0,6	0
192	Cala Panizo	16	Denia	9	232982,6	13	0	0,23516	0,8125	NA	NA
193	Cala Panizo	16	Moraira	10	220716,5	22	0	0,38824	0,69412	0,2	0
194	Cala Panizo	16	Cala Panizo	16	0	1819	1819	NA	NA	NA	NA
195	Cala Panizo	16	Benicasim	4	341372,8	4	0	NA	NA	NA	NA
196	Cala Panizo	16	Cala Reona	14	93404,9	258	258	0,08475	0,25	1	0
197	Cala Panizo	16	Cala de las Pulgas	15	26140,6	927	927	0,2	0	0	0
198	Cala Panizo	16	Punta del Cocedor	13	98317,5	92	0	0,34545	0,81053	0	0
199	Cala Panizo	16	Sant Carles de la Ràpita	3	41146,1	2	0	0,65161	0,31429	NA	NA
200	Cala Panizo	16	El Playazo	17	57364,8	38	0	0,58333	0	0,2	0
201	Cala Panizo	16	Cala Sant Francesc	1	620040,4	0	0	0,62581	0,54	NA	NA
202	Cala Panizo	16	Vilanova i la Geltrú	2	525218,4	1	0	NA	NA	NA	NA
203	Cala Panizo	16	Cala Rijana	18	1648,9	13	0	0,57368	0,33333	0,53333	0,17143
204	Cala Panizo	16	Velilla	19	185782,7	19	0	0,65333	0,7	0,8	0,31111
205	Cala Panizo	16	Ibiza	8	320925,8	66	0	0,37313	NA	NA	NA
206	Cala Panizo	16	Nerja	20	204631,7	40	0	0,62149	0,7	1	0
207	Cala Panizo	16	Cap d'Artrutx	6	561138,2	11	0	NA	NA	NA	NA
208	Cala Panizo	16	Punta Prima	5	589992,4	10	0	0,30323	0,11111	NA	NA
209	Cala Panizo	16	Mallorca	7	465061,6	12	0	NA	NA	NA	NA
210	Cala Panizo	16	Ghansou	21	27620,6	2	0	0,67213	0,86667	NA	NA
211	El Playazo	17	Santa Pola	12	198133,8	38	0	0,92329	0,49	0,90204	0,04545
212	El Playazo	17	La illeta	11	22542,7	8	0	0,98961	0,86316	0,56364	0
213	El Playazo	17	Denia	9	288758,2	11	0	0,95362	0,82667	NA	NA
214	El Playazo	17	Moraira	10	27587,6	23	0	0,98857	0,7	0,2	0
215	El Playazo	17	Benicasim	4	398718,2	5	0	NA	NA	NA	NA
216	El Playazo	17	Cala Reona	14	141887,9	91	0	0,38462	0,13333	0,92	0
217	El Playazo	17	Cala de las Pulgas	15	82115,2	869	869	1	0	0,2	0,22857
218	El Playazo	17	Punta del Cocedor	13	14993,1	52	0	0,97867	0,82222	0,2	0
219	El Playazo	17	Sant Carles de la Ràpita	3	468823,7	1	0	0,9746	0,26667	NA	NA
220	El Playazo	17	Cala Panizo	16	57364,8	338	338	0,58333	0	0,2	0
221	El Playazo	17	El Playazo	17	0	2727	2727	NA	NA	NA	NA
222	El Playazo	17	Cala Sant Francesc	1	676743,4	0	0	0,95224	0,53333	NA	NA
223	El Playazo	17	Vilanova i la Geltrú	2	582366,1	0	0	NA	NA	NA	NA
224	El Playazo	17	Cala Rijana	18	124867,4	66	0	0,6	0,2	0,47273	0,2
225	El Playazo	17	Velilla	19	148509,8	54	0	0,98824	0,76364	0,68	0,35
226	El Playazo	17	Ibiza	8	373379,2	40	0	0,98639	NA	NA	NA
227	El Playazo	17	Nerja	20	168334,1	73	0	0,95639	0,76364	0,68	0,05
228	El Playazo	17	Cap d'Artrutx	6	611888,3	9	0	NA	NA	NA	NA
229	El Playazo	17	Punta Prima	5	639356,8	4	0	0,97746	0,15556	NA	NA
230	El Playazo	17	Mallorca	7	514981,6	11	0	NA	NA	NA	NA
231	El Playazo	17	Ghansou	21	219009,5	8	0	1	0,9	NA	NA
232	Cala Sant Francesc	1	Santa Pola	12	479613,8	25	0	0,84561	0,47416	NA	NA
233	Cala Sant Francesc	1	La illeta	11	451669,4	6	0	0,93333	0,81849	NA	NA
234	Cala Sant Francesc	1	Denia	9	389077,4	28	0	0,88571	0,81217	NA	NA
235	Cala Sant Francesc	1	Moraira	10	403302,4	23	0	0,92727	0,6717	NA	NA
236	Cala Sant Francesc	1	Benicasim	4	291579,6	9	0	NA	NA	NA	NA
237	Cala Sant Francesc	1	Cala Reona	14	542745,1	68	0	0,70462	0,55	NA	NA
238	Cala Sant Francesc	1	Cala de las Pulgas	15	594652,6	4	0	0,94483	0,50909	NA	NA
239	Cala Sant Francesc	1	Punta del Cocedor	13	531171,8	32	0	0,91724	0,78644	NA	NA
240	Cala Sant Francesc	1	Sant Carles de la Ràpita	3	225093,1	1	0	0,63077	0,1	NA	NA
241	Cala Sant Francesc	1	Cala Panizo	16	620040,4	4	0	0,62581	0,54	NA	NA
242	Cala Sant Francesc	1	El Playazo	17	676743,4	7	0	0,95224	0,53333	NA	NA
243	Cala Sant Francesc	1	Cala Sant Francesc	1	0	1895	1895	NA	NA	NA	NA
244	Cala Sant Francesc	1	Vilanova i la Geltrú	2	10227,1	6	0	NA	NA	NA	NA
245	Cala Sant Francesc	1	Cala Rijana	18	768417,5	0	0	0,93239	0,62857	NA	NA
246	Cala Sant Francesc	1	Velilla	19	78204,1	1	0	0,93103	0,82	NA	NA
247	Cala Sant Francesc	1	Ibiza	8	326147,1	25	0	0,92941	NA	NA	NA
248	Cala Sant Francesc	1	Nerja	20	796161,7	0	0	0,89204	0,82	NA	NA
249	Cala Sant Francesc	1	Cap d'Artrutx	6	213033,1	30	0	NA	NA	NA	NA
250	Cala Sant Francesc	1	Punta Prima	5	241729,6	48	0	0,91579	0,4625	NA	NA
251	Cala Sant Francesc	1	Mallorca	7	257522,9	18	0	NA	NA	NA	NA
252	Cala Sant Francesc	1	Ghansou	21	895460,3	0	0	0,94386	0,896	NA	NA
253	Vilanova i la Geltrú	2	Santa Pola	12	387483,2	44	0	NA	NA	NA	NA
254	Vilanova i la Geltrú	2	La illeta	11	359097,7	13	0	NA	NA	NA	NA
255	Vilanova i la Geltrú	2	Denia	9	298310,8	43	0	NA	NA	NA	NA
256	Vilanova i la Geltrú	2	Moraira	10	313553,8	23	0	NA	NA	NA	NA
257	Vilanova i la Geltrú	2	Vilanova i la Geltrú	2	0	253	253	NA	NA	NA	NA
258	Vilanova i la Geltrú	2	Benicasim	4	190686,2	10	0	NA	NA	NA	NA
259	Vilanova i la Geltrú	2	Cala Reona	14	452442,5	73	0	NA	NA	NA	NA
260	Vilanova i la Geltrú	2	Cala de las Pulgas	15	500327,3	5	0	NA	NA	NA	NA
261	Vilanova i la Geltrú	2	Punta del Cocedor	13	440036,1	33	0	NA	NA	NA	NA
262	Vilanova i la Geltrú	2	Sant Carles de la Ràpita	3	122984,3	2	0	NA	NA	NA	NA
263	Vilanova i la Geltrú	2	Cala Panizo	16	525218,4	1	0	NA	NA	NA	NA
264	Vilanova i la Geltrú	2	El Playazo	17	582366,1	1	0	NA	NA	NA	NA
265	Vilanova i la Geltrú	2	Cala Sant Francesc	1	10227,1	2	0	NA	NA	NA	NA
266	Vilanova i la Geltrú	2	Cala Rijana	18	669728,8	0	0	NA	NA	NA	NA
267	Vilanova i la Geltrú	2	Velilla	19	68262,3	0	0	NA	NA	NA	NA
268	Vilanova i la Geltrú	2	Ibiza	8	251620,8	59	0	NA	NA	NA	NA
269	Vilanova i la Geltrú	2	Nerja	20	696299,2	0	0	NA	NA	NA	NA
270	Vilanova i la Geltrú	2	Cap d'Artrutx	6	227121,8	75	0	NA	NA	NA	NA
271	Vilanova i la Geltrú	2	Punta Prima	5	26534,6	29	0	NA	NA	NA	NA
272	Vilanova i la Geltrú	2	Mallorca	7	229812,7	30	0	NA	NA	NA	NA
273	Vilanova i la Geltrú	2	Ghansou	21	801374,7	0	0	NA	NA	NA	NA
274	Cala Rijana	18	Santa Pola	12	303284,3	18	0	0,90649	0,56667	0,8129	0,29091
275	Cala Rijana	18	La illeta	11	327371,1	8	0	0,97037	0,94118	0,45373	0,3
276	Cala Rijana	18	Denia	9	390104,9	5	0	0,93425	0,92308	NA	NA
277	Cala Rijana	18	Moraira	10	379737,8	19	0	0,96757	0,77143	0,21887	0,3
278	Cala Rijana	18	Benicasim	4	47991,9	3	0	NA	NA	NA	NA
279	Cala Rijana	18	Cala Reona	14	258230,6	47	0	0,41333	0	0,75294	0,24
280	Cala Rijana	18	Cala de las Pulgas	15	19020,8	44	0	0,97808	0,3	0,53333	0,45
281	Cala Rijana	18	Punta del Cocedor	13	262904,4	14	0	0,95949	0,9	0,53333	0,3
282	Cala Rijana	18	Sant Carles de la Ràpita	3	549995,7	0	0	0,95224	0,3	NA	NA

Continue to the next page

Continue from the previous page

id	Source Locality	Source ID	Sink locality	Sink ID	Geographic distance (metres)	Propagules (raw value)	Propagules (effective)	Fst value COI (*quadricollis*)	Fst value Wingless (*quadricollis*)	Fst value COI (*lejolisii*)	Fst value Wingless (*lejolsii*)
283	Cala Rijana	18	Cala Panizo	16	1648,9	54	0	0,57368	0,33333	0,53333	0,17143
284	Cala Rijana	18	El Playazo	17	124867,4	27	0	0,6	0,2	0,47273	0,2
285	Cala Rijana	18	Cala Sant Francesc	1	768417,5	0	0	0,93239	0,62857	NA	NA
286	Cala Rijana	18	Vilanova i la Geltrú	2	669728,8	1	0	NA	NA	NA	NA
287	Cala Rijana	18	Cala Rijana	18	0	229	229	NA	NA	NA	NA
288	Cala Rijana	18	Velilla	19	24611,4	834	834	0,96471	0,92308	0,17143	0
289	Cala Rijana	18	Ibiza	8	483338,8	27	0	0,96645	NA	NA	NA
290	Cala Rijana	18	Nerja	20	4430,5	96	0	0,93366	0,92308	0,16	0,34286
291	Cala Rijana	18	Cap d'Artrutx	6	724463,1	12	0	NA	NA	NA	NA
292	Cala Rijana	18	Punta Prima	5	754154,4	3	0	0,95733	0,33333	NA	NA
293	Cala Rijana	18	Mallorca	7	629071,5	12	0	NA	NA	NA	NA
294	Cala Rijana	18	Ghansou	21	16771,3	46	0	0,97681	1	NA	NA
295	Velilla	19	Santa Pola	12	321661,7	23	0	0,87771	0,58667	0,88571	0,36923
296	Velilla	19	La illeta	11	34492,4	10	0	0,96735	0,88889	0,52	0,46667
297	Velilla	19	Denia	9	40710,2	5	0	0,92899	0,85714	NA	NA
298	Velilla	19	Moraira	10	397317,1	17	0	0,97143	0,77895	0,3	0,46667
299	Velilla	19	Benicasim	4	492268,7	2	0	NA	NA	NA	NA
300	Velilla	19	Cala Reona	14	279163,4	55	0	0,74545	0,85714	0,88571	0,33333
301	Velilla	19	Cala de las Pulgas	15	210569,4	20	0	0,98644	0,65455	0,8	0,52
302	Velilla	19	Punta del Cocedor	13	283132,6	21	0	0,95102	0,84706	0,8	0,46667
303	Velilla	19	Sant Carles de la Ràpita	3	561987,7	1	0	0,96066	0,77647	NA	NA
304	Velilla	19	Cala Panizo	16	185782,7	18	0	0,65333	0,7	0,8	0,31111
305	Velilla	19	El Playazo	17	148509,8	16	0	0,98824	0,76364	0,68	0,35
306	Velilla	19	Cala Sant Francesc	1	78204,1	0	0	0,93103	0,82	NA	NA
307	Velilla	19	Vilanova i la Geltrú	2	68262,3	0	0	NA	NA	NA	NA
308	Velilla	19	Cala Rijana	18	24611,4	451	451	0,96471	0,92308	0,17143	0
309	Velilla	19	Velilla	19	0	1774	1774	NA	NA	NA	NA
310	Velilla	19	Ibiza	8	501846,7	31	0	0,96975	NA	NA	NA
311	Velilla	19	Nerja	20	19874,8	138	0	0,03478	0	0,2	0,44444
312	Velilla	19	Cap d'Artrutx	6	743198,4	8	0	NA	NA	NA	NA
313	Velilla	19	Punta Prima	5	77347,1	10	0	0,95714	0,3	NA	NA
314	Velilla	19	Mallorca	7	648332,2	8	0	NA	NA	NA	NA
315	Velilla	19	Ghansou	21	174421,2	10	0	0,2	0,85714	NA	NA
316	Ibiza	8	Santa Pola	12	18018,6	229	229	NA	NA	NA	NA
317	Ibiza	8	La illeta	11	158187,1	214	214	NA	NA	NA	NA
318	Ibiza	8	Denia	9	101881,4	238	238	NA	NA	NA	NA
319	Ibiza	8	Moraira	10	106873,8	203	203	NA	NA	NA	NA
320	Ibiza	8	Benicasim	4	158506,1	200	200	NA	NA	NA	NA
321	Ibiza	8	Cala Reona	14	231898,8	238	238	NA	NA	NA	NA
322	Ibiza	8	Cala de las Pulgas	15	294831,7	128	0	NA	NA	NA	NA
323	Ibiza	8	Punta del Cocedor	13	223528,6	273	273	NA	NA	NA	NA
324	Ibiza	8	Sant Carles de la Ràpita	3	188933,1	123	0	NA	NA	NA	NA
325	Ibiza	8	Cala Panizo	16	320925,8	150	0	NA	NA	NA	NA
326	Ibiza	8	El Playazo	17	373379,2	72	0	NA	NA	NA	NA
327	Ibiza	8	Cala Sant Francesc	1	326147,1	91	0	NA	NA	NA	NA
328	Ibiza	8	Vilanova i la Geltrú	2	251620,8	126	0	NA	NA	NA	NA
329	Ibiza	8	Cala Rijana	18	483338,8	64	0	NA	NA	NA	NA
330	Ibiza	8	Velilla	19	501846,7	56	0	NA	NA	NA	NA
331	Ibiza	8	Ibiza	8	0	5997	5997	NA	NA	NA	NA
332	Ibiza	8	Nerja	20	51917,1	68	0	NA	NA	NA	NA
333	Ibiza	8	Cap d'Artrutx	6	241408,7	37	0	NA	NA	NA	NA
334	Ibiza	8	Punta Prima	5	273060,6	45	0	NA	NA	NA	NA
335	Ibiza	8	Mallorca	7	148547,6	56	0	NA	NA	NA	NA
336	Ibiza	8	Ghansou	21	586248,7	54	0	NA	NA	NA	NA
337	Nerja	20	Santa Pola	12	339050,5	21	0	0,83912	0,58667	0,93846	0,02857
338	Nerja	20	La illeta	11	361817,4	8	0	0,92525	0,88889	0,55556	0,4
339	Nerja	20	Denia	9	423602,2	2	0	0,89267	0,85714	NA	NA
340	Nerja	20	Moraira	10	414170,9	13	0	0,93451	0,77895	0,31429	0,4
341	Nerja	20	Benicasim	4	505724,6	4	0	NA	NA	NA	NA
342	Nerja	20	Cala Reona	14	297920,3	52	0	0,71579	0,85714	1	0,05714
343	Nerja	20	Cala de las Pulgas	15	229136,7	23	0	0,95126	0,65455	1	0
344	Nerja	20	Punta del Cocedor	13	301543,3	28	0	0,90909	0,84706	1	0,4
345	Nerja	20	Sant Carles de la Ràpita	3	575135,9	1	0	0,92437	0,77647	NA	NA
346	Nerja	20	Cala Panizo	16	204631,7	12	0	0,62149	0,7	1	0
347	Nerja	20	El Playazo	17	168334,1	12	0	0,95639	0,76364	0,68	0,05
348	Nerja	20	Cala Sant Francesc	1	796161,7	0	0	0,89204	0,82	NA	NA
349	Nerja	20	Vilanova i la Geltrú	2	696299,2	0	0	NA	NA	NA	NA
350	Nerja	20	Cala Rijana	18	4430,5	869	869	0,93366	0,92308	0,16	0,34286
351	Nerja	20	Velilla	19	19874,8	1303	1303	0,03478	0	0,2	0,44444
352	Nerja	20	Ibiza	8	51917,1	28	0	0,935	NA	NA	NA
353	Nerja	20	Nerja	20	0	918	918	NA	NA	NA	NA
354	Nerja	20	Cap d'Artrutx	6	760575,2	2	0	NA	NA	NA	NA
355	Nerja	20	Punta Prima	5	791181,2	11	0	0,92035	0,3	NA	NA
356	Nerja	20	Mallorca	7	666037,5	6	0	NA	NA	NA	NA
357	Nerja	20	Ghansou	21	17872,6	32	0	0,17143	0,85714	NA	NA
358	Cap d'Artrutx	6	Santa Pola	12	421550,9	22	0	NA	NA	NA	NA
359	Cap d'Artrutx	6	La illeta	11	399302,3	14	0	NA	NA	NA	NA
360	Cap d'Artrutx	6	Denia	9	33983,8	36	0	NA	NA	NA	NA
361	Cap d'Artrutx	6	Moraira	10	347335,3	22	0	NA	NA	NA	NA
362	Cap d'Artrutx	6	Benicasim	4	319377,2	23	0	NA	NA	NA	NA
363	Cap d'Artrutx	6	Cala Reona	14	470031,7	15	0	NA	NA	NA	NA
364	Cap d'Artrutx	6	Cala de las Pulgas	15	535174,9	8	0	NA	NA	NA	NA
365	Cap d'Artrutx	6	Punta del Cocedor	13	463017,9	18	0	NA	NA	NA	NA
366	Cap d'Artrutx	6	Sant Carles de la Ràpita	3	287584,8	31	0	NA	NA	NA	NA
367	Cap d'Artrutx	6	Cala Panizo	16	561138,2	7	0	NA	NA	NA	NA
368	Cap d'Artrutx	6	El Playazo	17	611888,3	9	0	NA	NA	NA	NA
369	Cap d'Artrutx	6	Cala Sant Francesc	1	213033,1	94	0	NA	NA	NA	NA
370	Cap d'Artrutx	6	Vilanova i la Geltrú	2	227121,8	71	0	NA	NA	NA	NA
371	Cap d'Artrutx	6	Cala Rijana	18	724463,1	3	0	NA	NA	NA	NA
372	Cap d'Artrutx	6	Velilla	19	743198,4	4	0	NA	NA	NA	NA
373	Cap d'Artrutx	6	Ibiza	8	241408,7	23	0	NA	NA	NA	NA
374	Cap d'Artrutx	6	Nerja	20	760575,2	4	0	NA	NA	NA	NA
375	Cap d'Artrutx	6	Cap d'Artrutx	6	0	1788	1788	NA	NA	NA	NA
376	Cap d'Artrutx	6	Punta Prima	5	40902,4	37	0	NA	NA	NA	NA

Continue to the next page

Continue from the previous page

id	Source Locality	Source ID	Sink locality	Sink ID	Geographic distance (metres)	Propagules (raw value)	Propagules (effective)	Fst value COI (*quadricollis*)	Fst value Wingless (*quadricollis*)	Fst value COI (*lejolisii*)	Fst value Wingless (*lejolsii*)
377	Cap d'Artrutx	6	Mallorca	7	97864,4	13	0	NA	NA	NA	NA
378	Cap d'Artrutx	6	Ghansou	21	819503,2	2	0	NA	NA	NA	NA
379	Punta Prima	5	Santa Pola	12	452327,9	22	0	0,28	0,2	NA	NA
380	Punta Prima	5	La illeta	11	431222,1	14	0	0,85	0,5	NA	NA
381	Punta Prima	5	Denia	9	373309,6	22	0	0,4	0,3	NA	NA
382	Punta Prima	5	Moraira	10	379800,8	25	0	0,73333	0,29231	NA	NA
383	Punta Prima	5	Benicasim	4	359277,3	31	0	NA	NA	NA	NA
384	Punta Prima	5	Cala Reona	14	497876,9	24	0	0,62553	0,3	NA	NA
385	Punta Prima	5	Cala de las Pulgas	15	564219,9	8	0	0,8	0,12	NA	NA
386	Punta Prima	5	Punta del Cocedor	13	491677,9	15	0	0,77143	0,41538	NA	NA
387	Punta Prima	5	Sant Carles de la Ràpita	3	328473,6	32	0	0,94576	0,32308	NA	NA
388	Punta Prima	5	Cala Panizo	16	589992,4	13	0	0,30323	0,11111	NA	NA
389	Punta Prima	5	El Playazo	17	639356,8	12	0	0,97746	0,15556	NA	NA
390	Punta Prima	5	Cala Sant Francesc	1	241729,6	68	0	0,91579	0,4625	NA	NA
391	Punta Prima	5	Vilanova i la Geltrú	2	26534,6	46	0	NA	NA	NA	NA
392	Punta Prima	5	Cala Rijana	18	754154,4	5	0	0,95733	0,33333	NA	NA
393	Punta Prima	5	Velilla	19	77347,1	5	0	0,95714	0,3	NA	NA
394	Punta Prima	5	Ibiza	8	273060,6	25	0	0,6	NA	NA	NA
395	Punta Prima	5	Nerja	20	791181,2	7	0	0,92035	0,3	NA	NA
396	Punta Prima	5	Cap d'Artrutx	6	40902,4	20	0	NA	NA	NA	NA
397	Punta Prima	5	Punta Prima	5	0	685	685	NA	NA	NA	NA
398	Punta Prima	5	Mallorca	7	125144,3	18	0	NA	NA	NA	NA
399	Punta Prima	5	Ghansou	21	843701,5	4	0	0,97091	0,6	NA	NA
400	Mallorca	7	Santa Pola	12	327226,5	21	0	NA	NA	NA	NA
401	Mallorca	7	La illeta	11	306458,2	17	0	NA	NA	NA	NA
402	Mallorca	7	Denia	9	249771,5	20	0	NA	NA	NA	NA
403	Mallorca	7	Moraira	10	255421,1	24	0	NA	NA	NA	NA
404	Mallorca	7	Benicasim	4	256736,6	7	0	NA	NA	NA	NA
405	Mallorca	7	Cala Reona	14	373272,6	16	0	NA	NA	NA	NA
406	Mallorca	7	Cala de las Pulgas	15	439233,1	12	0	NA	NA	NA	NA
407	Mallorca	7	Punta del Cocedor	13	366771,7	8	0	NA	NA	NA	NA
408	Mallorca	7	Sant Carles de la Ràpita	3	244911,4	17	0	NA	NA	NA	NA
409	Mallorca	7	Cala Panizo	16	465061,6	3	0	NA	NA	NA	NA
410	Mallorca	7	El Playazo	17	514981,6	8	0	NA	NA	NA	NA
411	Mallorca	7	Cala Sant Francesc	1	257522,9	3	0	NA	NA	NA	NA
412	Mallorca	7	Vilanova i la Geltrú	2	229812,7	13	0	NA	NA	NA	NA
413	Mallorca	7	Cala Rijana	18	629071,5	6	0	NA	NA	NA	NA
414	Mallorca	7	Velilla	19	648332,2	5	0	NA	NA	NA	NA
415	Mallorca	7	Ibiza	8	148547,6	22	0	NA	NA	NA	NA
416	Mallorca	7	Nerja	20	666037,5	5	0	NA	NA	NA	NA
417	Mallorca	7	Cap d'Artrutx	6	97864,4	41	0	NA	NA	NA	NA
418	Mallorca	7	Punta Prima	5	125144,3	115	0	NA	NA	NA	NA
419	Mallorca	7	Mallorca	7	0	2152	2152	NA	NA	NA	NA
420	Mallorca	7	Ghansou	21	721687,7	4	0	NA	NA	NA	NA
421	Ghansou	21	Santa Pola	12	416061,4	19	0	NA	NA	NA	NA
422	Ghansou	21	La illeta	11	443821,9	11	0	NA	NA	NA	NA
423	Ghansou	21	Denia	9	506788,2	9	0	NA	NA	NA	NA
424	Ghansou	21	Moraira	10	493284,1	11	0	NA	NA	NA	NA
425	Ghansou	21	Benicasim	4	616870,6	7	0	NA	NA	NA	NA
426	Ghansou	21	Cala Reona	14	355388,2	57	0	NA	NA	NA	NA
427	Ghansou	21	Cala de las Pulgas	15	301084,8	18	0	NA	NA	NA	NA
428	Ghansou	21	Punta del Cocedor	13	365625,3	19	0	NA	NA	NA	NA
429	Ghansou	21	Sant Carles de la Ràpita	3	687173,3	1	0	NA	NA	NA	NA
430	Ghansou	21	Cala Panizo	16	27620,6	6	0	NA	NA	NA	NA
431	Ghansou	21	El Playazo	17	219009,5	21	0	NA	NA	NA	NA
432	Ghansou	21	Cala Sant Francesc	1	895460,3	0	0	NA	NA	NA	NA
433	Ghansou	21	Vilanova i la Geltrú	2	801374,7	0	0	NA	NA	NA	NA
434	Ghansou	21	Cala Rijana	18	16771,3	22	0	NA	NA	NA	NA
435	Ghansou	21	Velilla	19	174421,2	19	0	NA	NA	NA	NA
436	Ghansou	21	Ibiza	8	586248,7	38	0	NA	NA	NA	NA
437	Ghansou	21	Nerja	20	17872,6	4	0	NA	NA	NA	NA
438	Ghansou	21	Cap d'Artrutx	6	819503,2	10	0	NA	NA	NA	NA
439	Ghansou	21	Punta Prima	5	843701,5	8	0	NA	NA	NA	NA
440	Ghansou	21	Mallorca	7	721687,7	15	0	NA	NA	NA	NA
441	Ghansou	21	Ghansou	21	0	2993	2993	NA	NA	NA	NA
