# Supplementary material for: Oceanic currents maintain the genetic structure of non-marine coastal taxa in the western Mediterranean Sea
Source: NPJ Biodivers. 2023 Nov 20;2:25. doi: 10.1038/s44185-023-00028-0 (PMC11332052; doi:10.1038/s44185-023-00028-0)
Supplement: Supplementary file 2 — Reporting Summary [file 44185_2023_28_MOESM2_ESM.pdf]

nature portfolio

Corresponding author(s): DAPR 69de3f18-0cea-485b-9873-d55e4c3354d7

Last updated by author(s): May 25, 2023

Reporting Summary

Nature Portfolio wishes to improve the reproducibility of the work that we publish. This form provides structure for consistency and transparency in reporting. For further information on Nature Portfolio policies, see our Editorial Policies and the Editorial Policy Checklist.

Statistics

For all statistical analyses, confirm that the following items are present in the figure legend, table legend, main text, or Methods section.

n/a	Confirmed	
☐	☒	The exact sample size (*n*) for each experimental group/condition, given as a discrete number and unit of measurement
☒	☐	A statement on whether measurements were taken from distinct samples or whether the same sample was measured repeatedly
☐	☒	The statistical test(s) used AND whether they are one- or two-sided<br>*Only common tests should be described solely by name; describe more complex techniques in the Methods section.*
☒	☐	A description of all covariates tested
☒	☐	A description of any assumptions or corrections, such as tests of normality and adjustment for multiple comparisons
☒	☐	A full description of the statistical parameters including central tendency (e.g. means) or other basic estimates (e.g. regression coefficient) AND variation (e.g. standard deviation) or associated estimates of uncertainty (e.g. confidence intervals)
☐	☒	For null hypothesis testing, the test statistic (e.g. *F*, *t*, *r*) with confidence intervals, effect sizes, degrees of freedom and *P* value noted<br>*Give P values as exact values whenever suitable.*
☒	☐	For Bayesian analysis, information on the choice of priors and Markov chain Monte Carlo settings
☒	☐	For hierarchical and complex designs, identification of the appropriate level for tests and full reporting of outcomes
☒	☐	Estimates of effect sizes (e.g. Cohen's *d*, Pearson's *r*), indicating how they were calculated

Our web collection on statistics for biologists contains articles on many of the points above.

Software and code

Policy information about availability of computer code

Data collection	No software was used
Data analysis	R v4.2.2<br>DNAsp v6<br>PHASE as implemented in DNAsp v6<br>Ichthyop v3.361<br>QGIS v3.16.13

For manuscripts utilizing custom algorithms or software that are central to the research but not yet described in published literature, software must be made available to editors and reviewers. We strongly encourage code deposition in a community repository (e.g. GitHub). See the Nature Portfolio guidelines for submitting code & software for further information.

Data

Policy information about availability of data

All manuscripts must include a data availability statement. This statement should provide the following information, where applicable:

- Accession codes, unique identifiers, or web links for publicly available datasets
- A description of any restrictions on data availability
- For clinical datasets or third party data, please ensure that the statement adheres to our policy

The data is fully available in the Supplementary material.

Research involving human participants, their data, or biological material

Policy information about studies with human participants or human data. See also policy information about sex, gender (identity/presentation), and sexual orientation and race, ethnicity and racism.

Reporting on sex and gender	Not applicatable
Reporting on race, ethnicity, or other socially relevant groupings	Not applicatable
Population characteristics	Not applicable
Recruitment	Not applicable
Ethics oversight	Not applicable

Note that full information on the approval of the study protocol must also be provided in the manuscript.

Field-specific reporting

Please select the one below that is the best fit for your research. If you are not sure, read the appropriate sections before making your selection.

☐ Life sciences ☐ Behavioural & social sciences ☒ Ecological, evolutionary & environmental sciences

For a reference copy of the document with all sections, see nature.com/documents/nr-reporting-summary-flat.pdf

Ecological, evolutionary & environmental sciences study design

All studies must disclose on these points even when the disclosure is negative.

Study description	We combined previously published genetic information with a dispersal model of propagules using a multiyear simulation of the western Mediterranean Sea. We selected 20 locations across the Iberian Mediterranean Coast and 1 location from the Moroccan Mediterranean Coast. We considered seven years for our model, with 1,000 propagules released every two weeks.
Research sample	Our study model is the supratidal coastal beetles of the genus Ochthebius. We used a multi-year approach to cover any temporal fluctuation of abiotic conditions, releasing a high number of propagules every two weeks.<br>This information was compared with the most updated genetic dataset (Villastrigo et al., 2022), which includes data on two molecular markers (COI and wingless genes) and three specimens per locality. Limiting funding is the main problem when acquiring a larger dataset.
Sampling strategy	As the study organisms have multivoltine reproduction and exhibit a large number of individuals in each locality, we opted for a high number of propagules to be dispersed (1,000 propagules every two weeks).
Data collection	Not applicable
Timing and spatial scale	The biophysical model that we used included information for seven years between January 2009 and December 2015. Artificial propagules were released every two weeks to capture temporal variability.
Data exclusions	The molecular data from Villastrigo et al. (2022) were used in this study. We excluded localities for which only one individual was sequenced.
Reproducibility	The biophysical model can be used upon request by the authors (not by us). Detailed methodologies were included in the methodology section. All software used were open-source and available. The raw data are provided in the Supplementary Material.
Randomization	Propagules were randomly released every two weeks to capture all possible variability. In each locality, a buffer area of 50 meters

Randomization	was predefined and propagules were randomly liberated.
Blinding	Blinding was not relevant to our simulations, as propagules were released every two weeks for 7 years. Releases were randomly distributed in buffer areas per locality.

Did the study involve field work? ☐ Yes ☒ No

Reporting for specific materials, systems and methods

We require information from authors about some types of materials, experimental systems and methods used in many studies. Here, indicate whether each material, system or method listed is relevant to your study. If you are not sure if a list item applies to your research, read the appropriate section before selecting a response.

Materials & experimental systems

n/a	Involved in the study
☒	☐ Antibodies
☒	☐ Eukaryotic cell lines
☒	☐ Palaeontology and archaeology
☒	☐ Animals and other organisms
☒	☐ Clinical data
☒	☐ Dual use research of concern
☒	☐ Plants

Methods

n/a	Involved in the study
☒	☐ ChIP-seq
☒	☐ Flow cytometry
☒	☐ MRI-based neuroimaging
